# Supplementary material for: Definition of functionally and structurally distinct repressive states in the nuclear receptor PPARγ
Source: Nat Commun. 2019 Dec 20;10:5825. doi: 10.1038/s41467-019-13768-0 (PMC6925260; doi:10.1038/s41467-019-13768-0)
Supplement: Supplementary file 1 — Supplementary Information [file 41467_2019_13768_MOESM1_ESM.pdf]

Supplementary Information

# Definition of functionally and structurally distinct repressive states in the nuclear receptor PPARγ

Heidari et al.

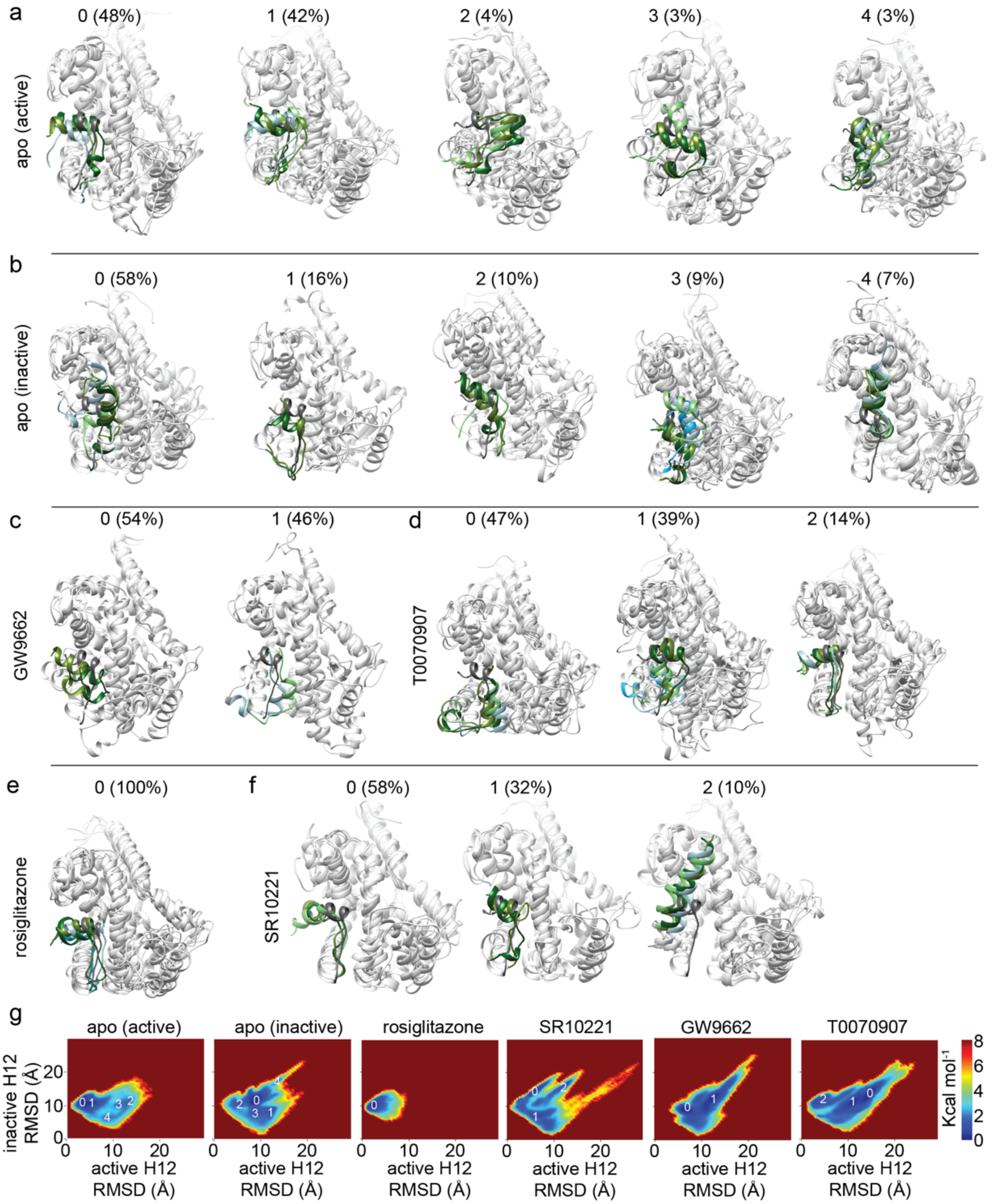


**Supplementary Figure 1. Accelerated MD reveals a more diverse structural ensemble for apo PPARγ and PPARγ bound to inverse agonists/antagonists compared to agonist bound PPARγ.** a-f) Representative structures from clustering based on helix 12 RMSD of structures in the wells indicated in panel g. The identity of the energy well and the calculated prevalence of each well's structures within the Boltzmann ensemble (see methods) is indicated by the number and percent in parentheses respectively. The prevalence of the structures within a particular well is indicated by the color of helix 12 ranging from dark green for the most prevalent to olive drab, light

green, light blue and then deep sky blue for the least prevalent. For reference, an active structure (rosiglitazone bound PPARγ; 2PRG chain A) is shown with helix 12 colored dim grey. g) The locations of the wells that were sampled to produce the representative structures in panels a-f are shown. Supplementary Table 7 shows the relative energies of the wells indicated in panel g.

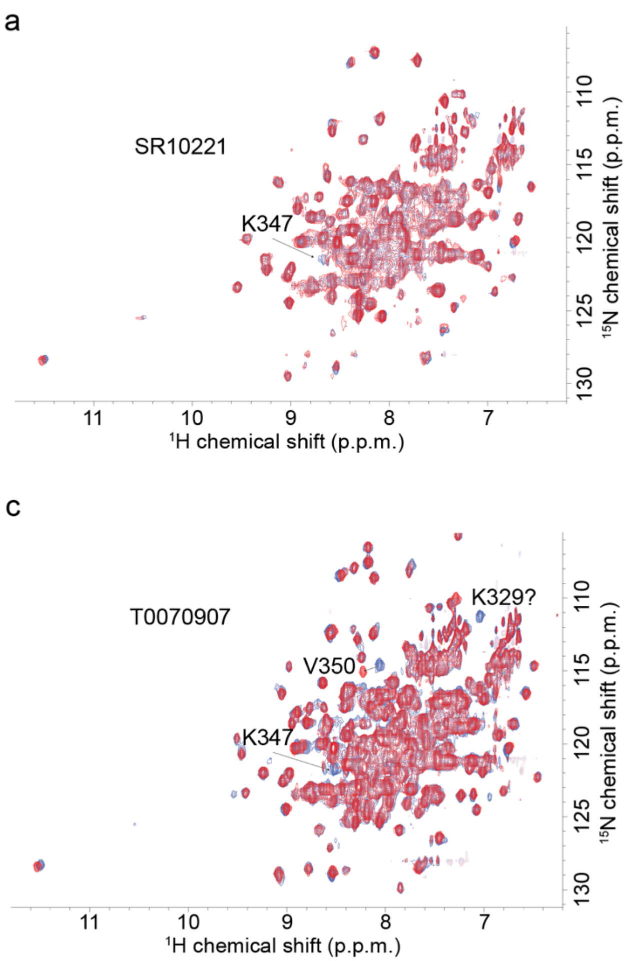


**Supplementary Figure 2. TROSY-HSQC of K347A mutants compared to wt PPARγ LBD.** a-c) Comparison of K347A mutant (red), that disrupts interaction between helix 4 and helix 12, and wt PPARγ LBD (blue) bound to the indicated ligands. Probable assignments, based on rosiglitazone bound PPARγ and apo PPARγ assignments, for residues affected by the mutation are indicated. The mutation consistently affects a resonance with shifts between 121 and 123 (15N) and 8.5 and 8.7 (1H) in all forms. Two rosiglitazone assigned residues are in this area, K347 and L463. L463 is far from the coregulator binding surface and we mutated K347, so we assume this resonance is K347.

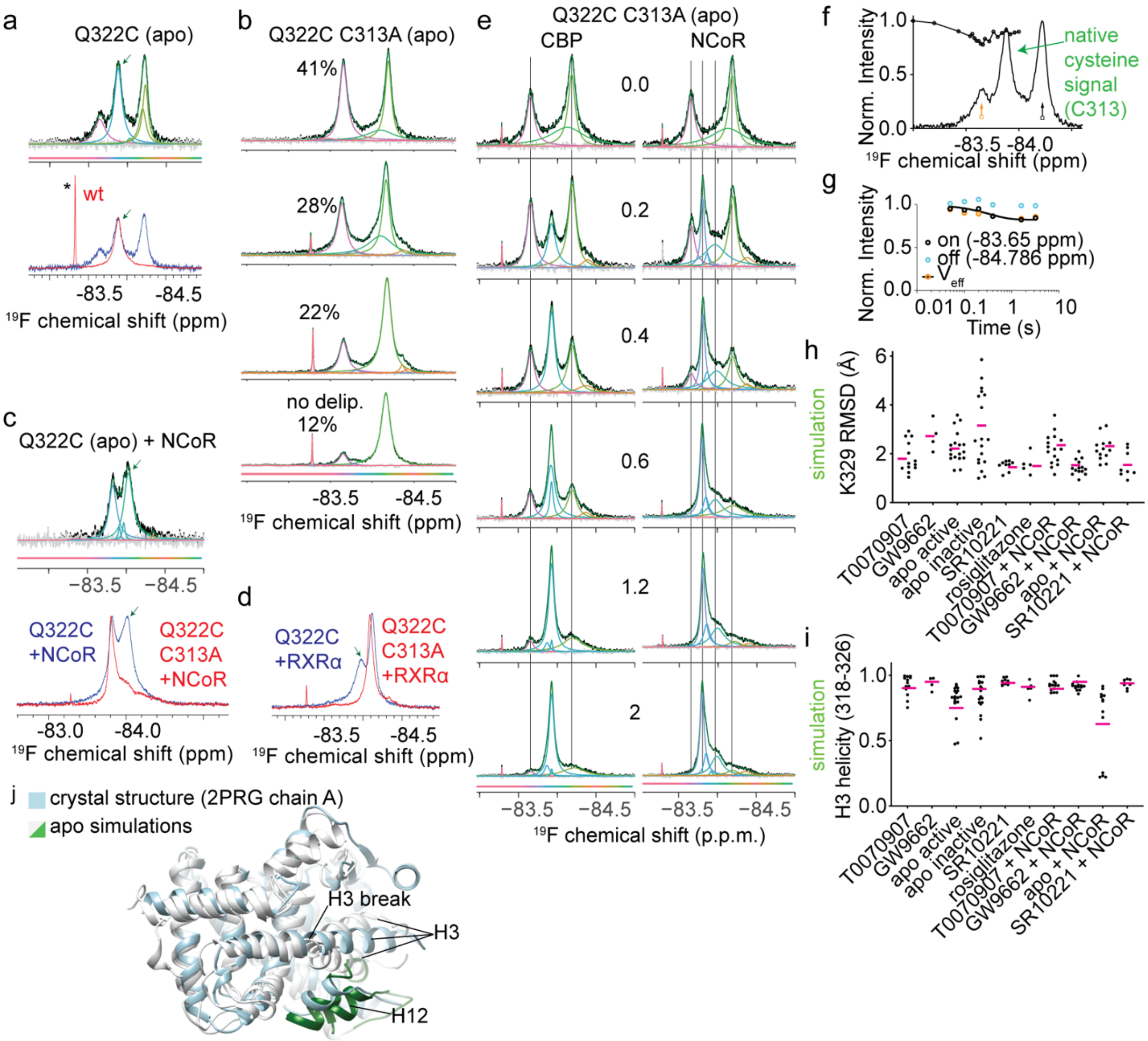


**Supplementary Figure 3. The charge clamp region of helix 3 is composed of a diverse structural ensemble in apo that consolidates upon binding coregulators, RXRα or ligands.** a) The spectrum of wt PPARγ-BTFA (red) overlays well with the middle peak of PPARγ$^{Q322C}$-BTFA, indicating that the middle peak originates from labeling of the sole native cysteine (C313). The green arrow indicates signal from C313. b) Examples of spectra from delipidated PPARγ$^{C313A,Q322C}$-BTFA from different purifications and from non-delipidated PPARγ$^{C313A,Q322C}$-BTFA (bottom panel). The delipidation efficacy is variable and affects the relative population of apo peaks with delipidation increasing the population of the left peak. This variability is also observed with spectra of apo bound to peptides. c-d) Overlay of PPARγ$^{Q322C}$-BTFA (blue), which can be labeled at the native cysteine (C313), and PPARγ$^{C313A,Q322C}$-BTFA (red) which cannot, bound to either c) NCoR or d) RXRα. Green arrows indicate signal from native cysteine (C313) labeling. e) Either CBP or NCoR was titrated into ligand free PPARγ$^{C313A,Q322C}$-BTFA (apo) at the indicated molar ratios of peptide to PPARγ. 0.0 indicates no peptide present. f) CEST indicates exchange between the peak denoted by the orange arrow and the black arrow, but not the central peak (native cysteine signal). The height of the circles represents the height of the right peak (black arrow) following a saturating pulse given at the location of the black circles. g) Saturating NMR pulses were applied at the orange arrow in panel f and an off-target shift equidistant from and to the right of the right peak (black arrow panel f). The height of the right peak (black arrow panel f) was measured after various durations of the saturating NMR pulse. The data were then fit to a single exponential, which indicates 0.5 $s^{-1}$ exchange (0.3-0.7 95% CI), however this is at the limit of detection and the exchange rate may be lower. h) Boltzmann averages and individual values of the RMSD of helix 3 charge clamp (K329) compared to PDB code 1PRG crystal structure. i) Boltzmann averages of the helicity of a section of helix 3 near the charge clamp that contains the Q322C-BTFA probe calculated using the cpptraj program. The helicity of all residues in the section were averaged for each simulation (black circles) and then the Boltzmann averages were calculated. j) Representative structures from active and inactive apo simulations (white and green) are shown compared to an active crystal structure (light blue; 2PRG chain A). Source data are provided as a Source Data file (Source data_Heidari.xlsx). The color of the deconvoluted peaks denotes chemical shift as shown by the color bar.

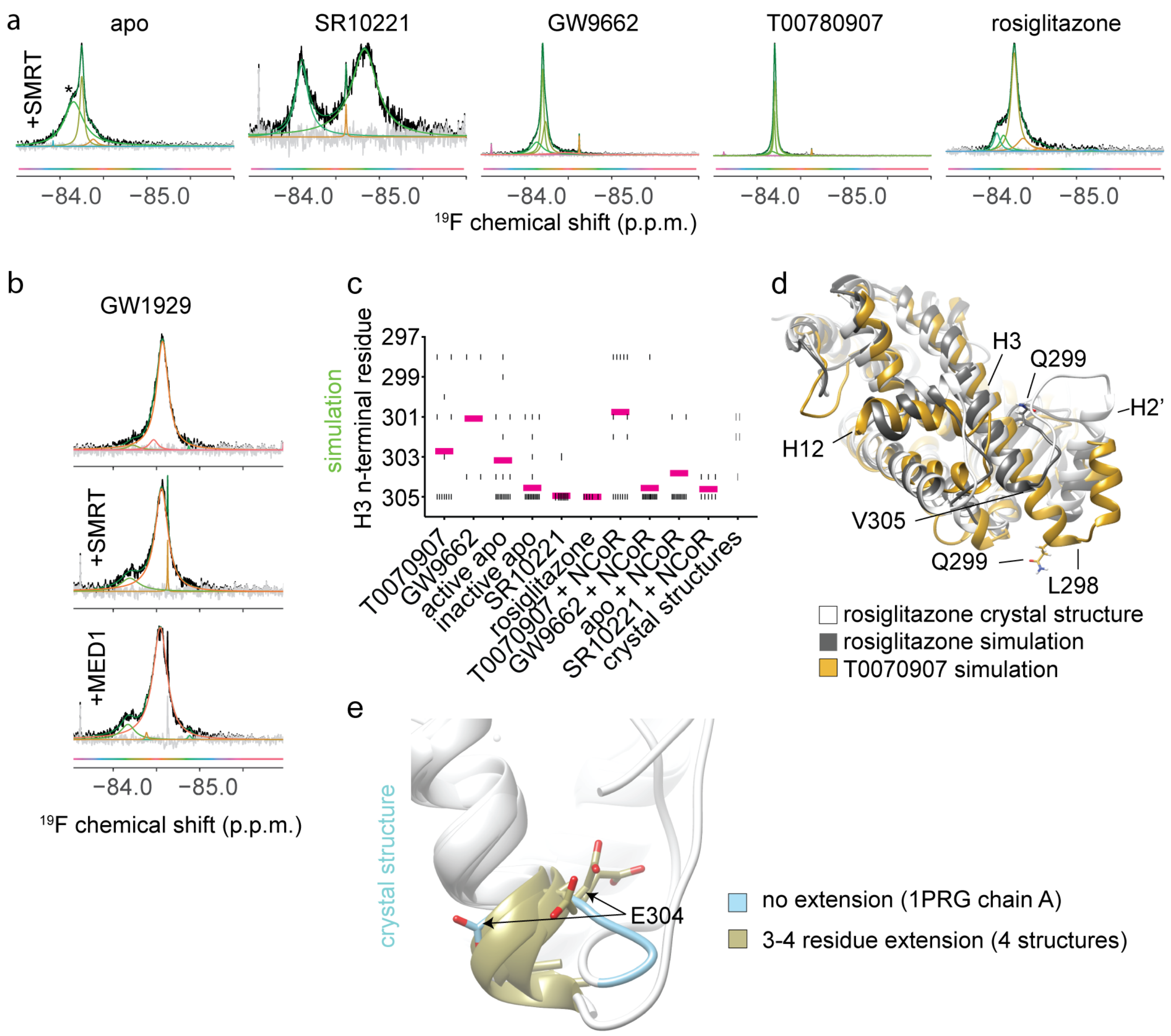


**Supplementary Figure 4. The omega loop shows diverse ligand dependent conformations.** a-b) PPARγ-Q299C was labeled with BTFA bound to the indicated ligands (or apo) and peptides and fluorine NMR spectra were collected. c) The extent of helix 3 extension beyond the normally observed n-terminus (V305) is indicated for individual simulations along with the Boltzmann average (pink bar). To calculate helix 3 extension, the dot product of the amide NH bond vectors of the normally observed n-terminus (V305) residue and the residues beyond that were calculated separately over time. Then each pair of residues that had dot product values less than 50 at least 60% of the time were considered to be part of the helical extension. d) Comparison of the crystal structure of rosiglitazone bound PPARγ (white; PDB code 2PRG) with simulation structures of rosiglitazone bound PPARγ (dim grey) and T0070907 bound PPARγ (gold). The location of the probe (Q299) and the usual n-terminus of helix 3 (V305) are shown. e) Four of 286 crystal structure chains show n-terminal helix 3 extension of 3-4 residues past V305 to residue 302 or 301 (khaki). In other PPARγ crystal structure chains helix 3 starts at V305 as represented by the light blue structure(PDB code 1PRG).The four chains with this 3-4 residue helix 3 n-terminal extension are PPARγ bound to a partial agonist (PDB code 1ZEO chain A) and PPARγ bound to two antagonists (6C5Q, 6C5T) and of an agonist (rosiglitazone) bound PPARγ (PDB code 2PRG chain B). The side chain of E304 is shown, one structure does not have a resolved E304 beyond the beta carbon. Source data are provided as a Source Data file (Source data_Heidari.xlsx). The color of the deconvoluted peaks denotes chemical shift as shown by the color bar.

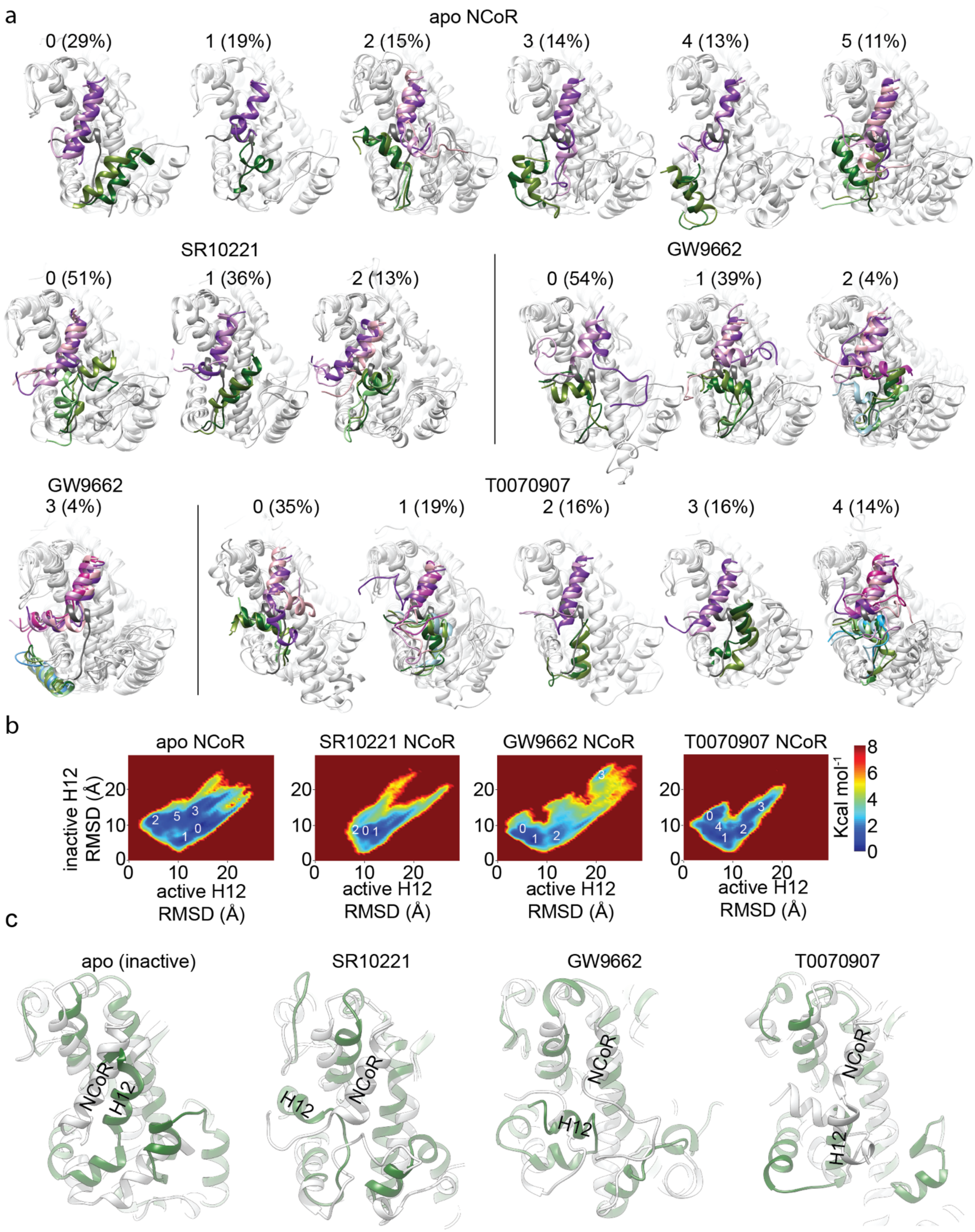


**Supplementary Figure 5. Accelerated molecular simulations indicate that two inverse agonists induce different conformations when bound to a CoRNR box peptide from the corepressor NCoR.** aMD simulations were run of PPARγ bound to the indicated ligands and the NCoR peptide. a) Representative structures from clustering based on helix 12 RMSD of structures in the wells indicated in panel b. The identity of the energy well

and the calculated prevalence of each well's structures within the Boltzmann ensemble (see methods) is indicated by the number and percent in parentheses respectively. The prevalence of the structures within a particular well is indicated by the color of helix 12 ranging from dark green for the most prevalent to olive drab, light green, light blue and then deep sky blue for the least prevalent. For reference, an active structure (rosiglitazone bound PPARγ; 2PRG chain A) is shown with helix 12 colored dim grey. The relative energies of the wells indicated in panel b are shown in Supplementary Table 7. c) Representative structures from the largest cluster of the lowest energy well were aligned for the indicated PPARγ LBD ligand complexes with (white) or without (green) bound NCoR. The location of the NCoR peptide and helix 12 (H12) of the PPARγ-ligand simulation without NCoR are indicated. All of these simulations were started with helix 12 in the inactive conformation.

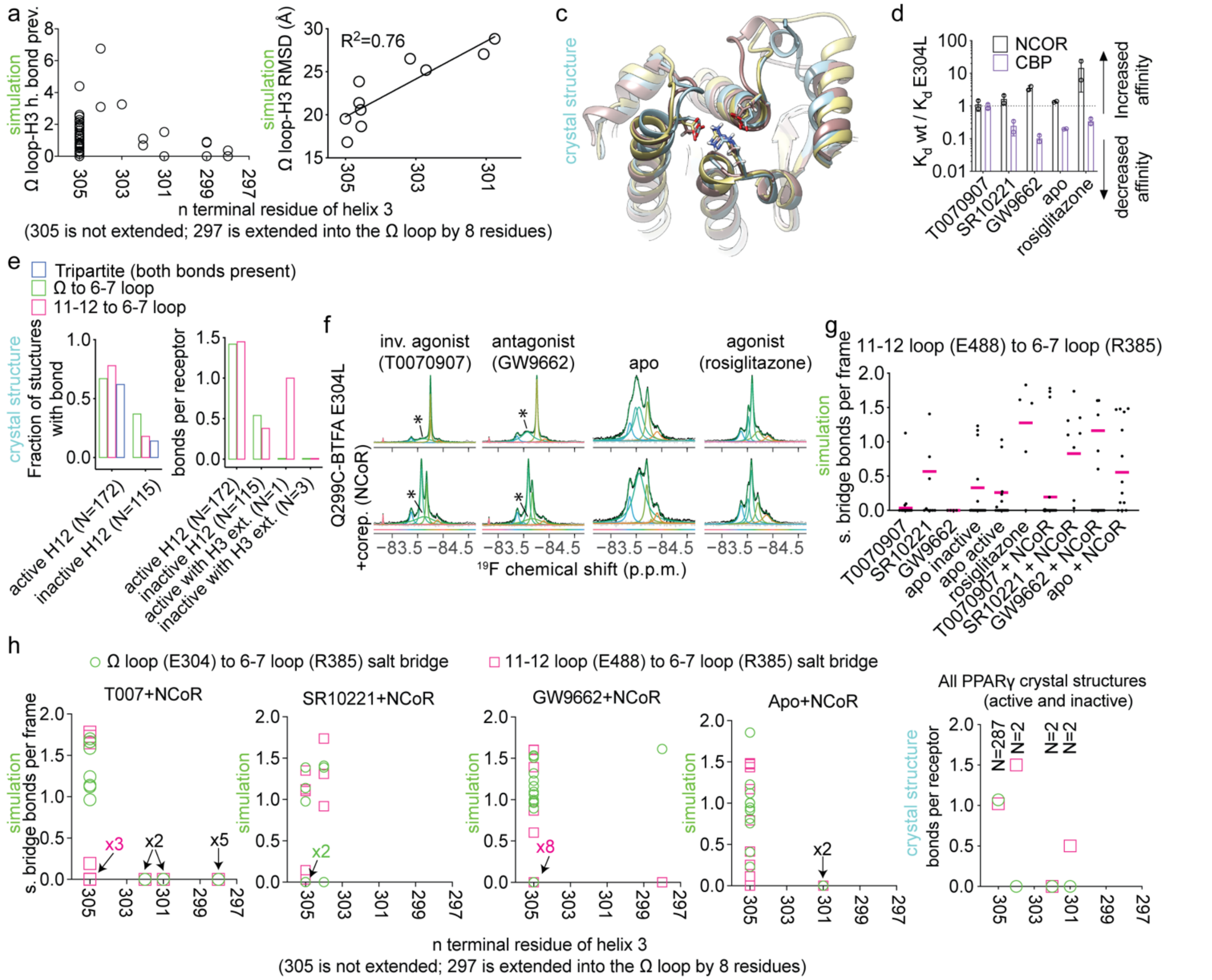


**Supplementary Figure 6. Helix 3 extension reduces omega loop-helix 3 interaction and salt-bridge interactions.** a) The total number of hydrogen bonds between the omega loop and helix 3 versus Boltzmann average helix 3 extension. b) The Boltzmann average of the root mean square deviation between helix 3 and the omega loop versus Boltzmann average helix 3 extension. c) Overlay of crystal structures of PPARγ (khaki, 2PRG), PPARα (light blue, 2ZNN) and PPARδ (rosy brown, 5U3Q). The tripartite salt bridge member residues for PPARγ (E304, R385 and E488), PPARα (E267, R348 and E451) and PPARδ (E240, R321, and E424) are shown. d) Effect of E304L mutation on CBP and NCoR peptide affinity for PPARγ-LBD bound to the indicated ligands. Dissociation constant ($K_d$) of the indicated peptide was measured using fluorescence anisotropy by titrating the PPARγ-ligand complex into 50nM Fluorescein labeled coregulator peptide. This was done using E304L with the 6x Histidine intact, while a similar experiment shown in Figure 7b utilized E304L without the 6x Histidine tag. The average (bar heights), standard deviation (error bars) and individual values (open circles) from two independent anisotropy experiments using protein from the same purification batch are shown. e) Analysis of the indicated salt bridge formation for all available crystal structures of PPARγ. f) $^{19}$F NMR of PPARγ$^{E304LQ299C}$-BTFA alone (apo) or bound to the indicated drugs and (lower panel) bound to a corepressor (NCoR) peptide. These data were obtained with protein from a separate protein purification batch than similar spectra shown in Figure 7d. Asterisks point to signal coming from apo/*E. coli* lipid bound Q299C-BTFA signal. This protein batch likely had more residual E. coli lipids bound which resulted in incomplete T0070907 and GW9662 covalent attachment to C313. The farthest left blue peak overlays with signal from labeled native cysteine (C313; see Supplementary Figure 3a). The farthest right peak (brown) may originate from misfolded protein or contaminating protein[1] g) Prevalence of the helix 6-7 loop (R385) to helix 11-12 loop (E488) salt-bridge is shown for the indicated complexes for individual cMD simulations started from representative structures from the lowest energy wells in the aMD potential energy landscapes (black dots). The Boltzmann average is shown by a magenta bar±±±.  h) Comparison of the prevalence of the indicated salt bridges and helical extension for the indicated complexes and for all available crystal structures. Source data are provided as a Source Data file (Source data_Heidari.xlsx). The color of the deconvoluted peaks denotes chemical shift as shown by the color bar.

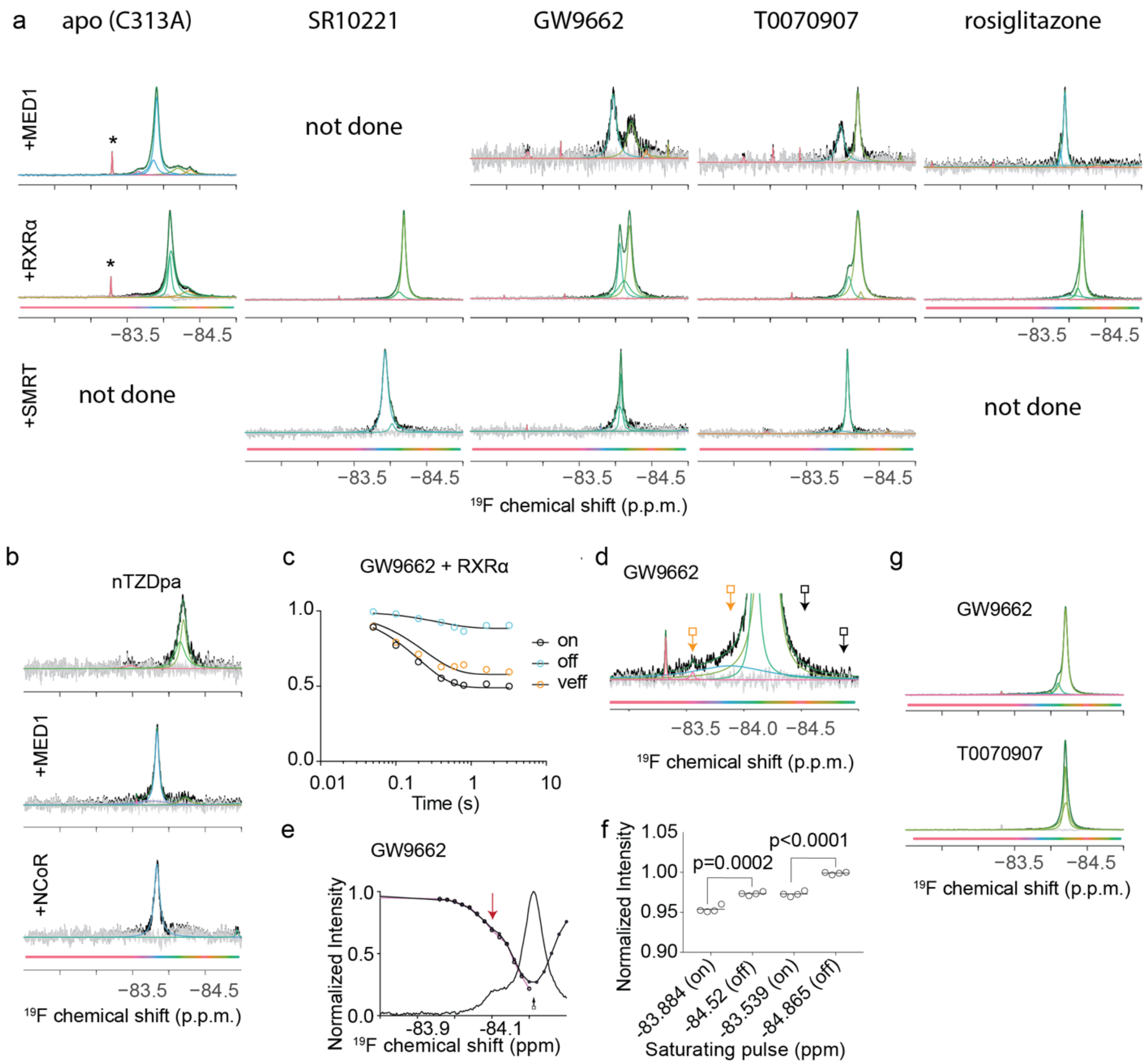


**Supplementary Figure 7. $^{19}$F NMR reveals structurally and functionally distinct states in the charge clamp region of helix 3.**

a, b and g) PPARγ$^{Q322C}$ was loaded with the indicated ligand and labeled with BTFA. For Apo, PPARγ$^{C313A,Q322C}$-BTFA was used. The indicated coregulators were added at a 2:1 molar ratio. 19F NMR spectra were acquired followed by deconvolution. c) Saturating pulses (circles) were applied at the minor peak chemical shift (on; black circles) and at a control location equidistant from the main peak (off; blue circles) for various durations to PPARγ$^{Q322C}$-BTFA bound to GW9662 and heterodimerized with RXRα. The normalized intensity of the main peak was monitored and plotted against the duration of the pulses and fit to determine the exchange rate (1.9 $s^{-1}$; 95% CI=1.7-2.2). d) Zoom in of the deconvolution of the GW9662 spectrum in panel g, showing downfield (left shifted) minor peaks. We tested for exchange of these peaks with the major peak using selective saturating pulses in this region (orange arrows) and control upfield pulses equidistant from the major peak (black arrows). The results of this test are shown in panel f. e) CEST indicates exchange between the minor left peak (orange arrow) and the major peak (black arrow) in PPARγ$^{Q322C}$-BTFA bound to GW9662. The position of the black circles indicate the region of saturation and their y position indicates the height of the major peak (black arrow) upon saturation. A slight deviation is noted when saturating near the minor peak (orange arrow) indicating exchange between the minor and major peak. f) The intensity of the major peak was measured after saturating pulses at the indicated locations (see panel d) and compared. This was repeated 4 times on the same sample at each location. A two-tailed unpaired t-test indicates that the downfield peaks exist and exchange with the major peak. -84.52 vs -83.884 p=0.0002; t=8.11; df=6; Raw means are 471351 and 481008; 95% CI of difference = 6743-12570. -84.865 vs -83.539; p=<0.0001; t=14.99; df=6; Raw means are 480786 and 493606; 95% CI of difference = 10727- 14912 g) Spectra of PPARγQ322C-BTFA bound to GW9662 or T0070907 using different samples from those shown in Figure 1. Source data are provided as a Source Data file (Source data_Heidari.xlsx). The color of the deconvoluted peaks denotes chemical shift as shown by the color bar.

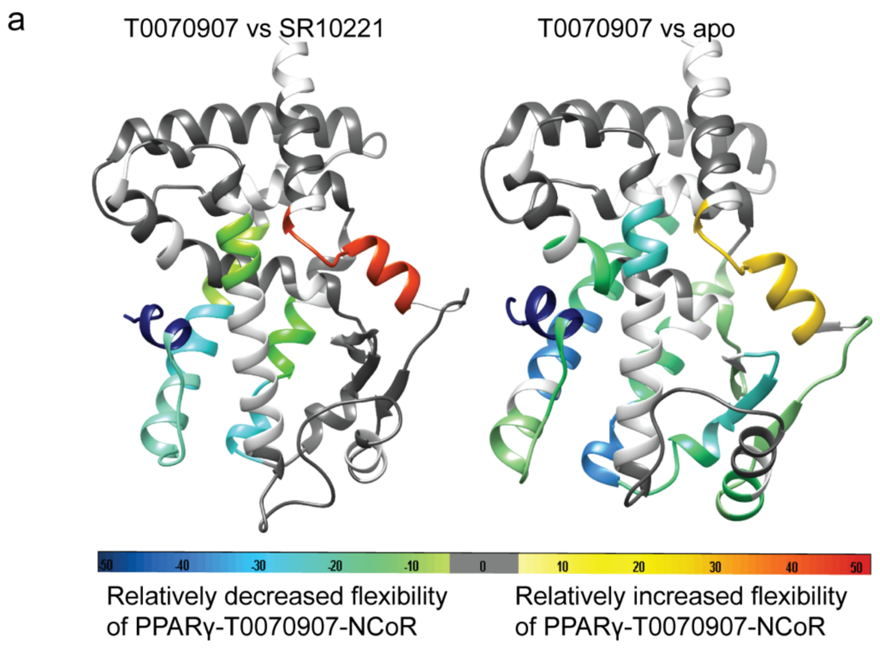


b

| sequence | charge | start | end | Apo vs T007 | 10221 vs T007 |
|---|---|---|---|---|---|
| RALAKHLYDS | 3 | 240 | 249 | 0 (2)* | 0 (2)* |
| RALAKHLYDSY | 3 | 240 | 250 | 0 (1)* | 0 (1)* |
| IKSFPLTKAKARAIL | 3 | 251 | 265 | 23 (5) | 39 (3) |
| TGKTTDKSPFVIYDM | 3 | 266 | 280 | -7 (2) | -2 (2)* |
| TGKTTDKSPFVIYDMNSLM | 3 | 266 | 284 | -10 (3) | |
| MGEDKIKFKHITPLQEQSKE | 3 | 285 | 304 | -3 (3)* | -1 (2)* |
| MGEDKIKFKHITPLQEQSKEVA | 3 | 285 | 306 | -4 (2)* | 0 (2)* |
| KIKFKHITPLQEQSKEVA | 3 | 289 | 306 | -3 (2)* | -1 (3)* |
| IRIFQGCQ | 2 | 307 | 314 | | 7 (3) |
| AVQEITE | 1 | 320 | 326 | -21 (2) | -6 (1) |
| YAKSIPGF | 2 | 327 | 334 | 0 (1)* | 0 (1)* |
| YAKSIPGFVNL | 2 | 327 | 337 | 0 (1)* | 1 (1)* |
| DLNDQVTL | 1 | 338 | 345 | -1 (1)* | -1 (1)* |
| LKYGVHE | 2 | 346 | 352 | -10 (1) | -8 (1) |
| LKYGVHEIIY | 2 | 346 | 355 | -4 (0)* | -2 (0)* |
| LKYGVHEIIYTM | 3 | 346 | 357 | -3 (1)* | -2 (1)* |
| ASLMNKDGVL | 2 | 359 | 368 | -8 (2) | -1 (1)* |
| MNKDGVL | 2 | 362 | 368 | -8 (2) | -2 (1)* |
| ISEGQGFMTRE | 2 | 369 | 379 | -22 (3) | 0 (2)* |
| ISEGQGFMTREFL | 2 | 369 | 381 | -16 (3) | 1 (2)* |
| FLKSLRKPFGD | 2 | 380 | 390 | -1 (1)* | 0 (2)* |
| FLKSLRKPFGDFMEPKFEF | 3 | 380 | 398 | -14 (2) | -4 (2)* |
| LRKPFGDF | 2 | 384 | 391 | -33 (3) | -22 (3) |
| LRKPFGDFMEPKFEF | 3 | 384 | 398 | -16 (3) | -8 (2) |
| AVKFNAL | 2 | 399 | 405 | -9 (2) | -3 (2)* |
| NALELDDSDL | 1 | 403 | 412 | 1 (1)* | 1 (1)* |
| VIILSGDRPGLL | 2 | 418 | 429 | 0 (2)* | 1 (2)* |
| IILSGDRPGLL | 2 | 419 | 429 | 0 (2)* | 1 (2)* |
| IILSGDRPGLLNVKPIE | 3 | 419 | 435 | 0 (2)* | 1 (2)* |
| IILSGDRPGLLNVKPIED | 3 | 419 | 436 | 0 (2)* | -1 (2)* |
| NVKPIEDIQDNL | 2 | 430 | 441 | 0 (1)* | 1 (1)* |
| NVKPIEDIQDNLLQA | 2 | 430 | 444 | 0 (1)* | 0 (1)* |
| LELQLKLNHPESSQL | 3 | 445 | 459 | 0 (1)* | 0 (1)* |
| ELQLKLNHPESSQL | 2 | 446 | 459 | -1 (2)* | 0 (2)* |
| QLKLNHPESSQL | 2 | 448 | 459 | 0 (1)* | 0 (1)* |
| KLNHPESSQL | 2 | 450 | 459 | -1 (2)* | -1 (2)* |
| FAKLLQKMTDL | 2 | 460 | 470 | -1 (1)* | -1 (2)* |
| FAKLLQKMTDLRQ | 3 | 460 | 472 | -9 (2) | -4 (2)* |
| LQKMTDL | 2 | 464 | 470 | -2 (2)* | -2 (2)* |
| LQKMTDLRQ | 3 | 464 | 472 | -7 (2) | -7 (3) |
| RQIVTE | 2 | 471 | 476 | -30 (3) | |
| RQIVTEHVQL | 3 | 471 | 480 | -32 (2) | -26 (2) |
| LQVIKKTETDM | 2 | 481 | 491 | -6 (2) | -2 (2)* |
| LQVIKKTETDMSLHPLL | 3 | 481 | 497 | -12 (2) | -10 (2) |
| LQVIKKTETDMSLHPLLQE | 3 | 481 | 499 | -18 (2) | -17 (2) |
| IKKTETDMSLHPLL | 3 | 484 | 497 | -11 (2) | -11 (2) |
| HPLLQEIYKDLY | 2 | 494 | 505 | -72 (4) | -80 (3) |
| QEIYKDLY | 1 | 498 | 505 | -62 (3) | -67 (3) |

**Supplementary Figure 8. HDX-MS data of PPARγ bound to NCoR peptide alone or co-bound to T0070907 or SR10221.** a) overlay of the HDX-MS data onto the crystal structure of PPARγ LBD. Colors match those in panel b, any areas without peptide coverage are white. b) Detected peptide sequences residue numbers and change in HDX. A decrease in exchange in the T0070907 complex peptide compared to the indicated complexes is shown as a negative number while an increase is shown as a positive number. Standard deviation is shown in parentheses. Peptides with significant differences are shown in color, while insignificant are shown in grey and have an asterisk. Blank white slots mean matching peptides were not found for that condition. Source data are provided as a Source Data file (Source data_Heidari.xlsx).

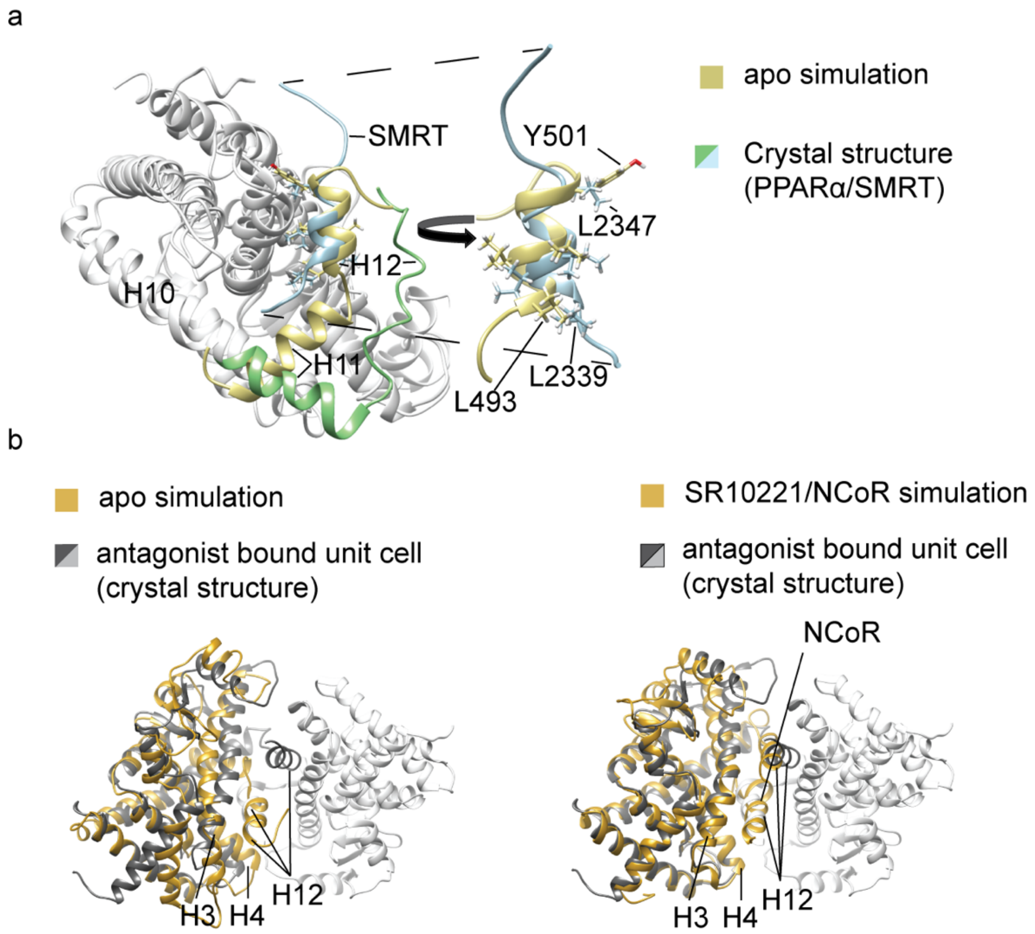


**Supplementary Figure 9. Simulations indicate a low-energy auto-repressed conformation exists for apo PPARγ.** a) A representative structure from a low energy apo well (white and gold) is compared to the crystal structure of PPARα (PDB code 1KKQ; white and green) bound to a CoRNR box peptide from the corepressor SMRT (light blue). Comparison of relative position of PPARγ helix 12 residues with SMRT residues is highlighted. b) A domain swapped dimer, is observed in antagonist bound PPARγ (PDB 6c5q), where helix 12 from one monomer of the crystallographic unit cell binds to the coregulator binding surface of another unit cell monomer (light and dark grey). This helix 12-coregulator binding surface interaction is similar to that observed in some low-energy simulation generated structures. Representative structures from the fifth lowest energy well in apo PPARγ where helix 12 binds to the coregulator binding surface (left; gold) and the second lowest energy well from the PPARγ cobound to SR10221 and NCoR (right; gold) simulations are aligned with the unit cell of an antagonist bound structure (PDB code 6c5q).

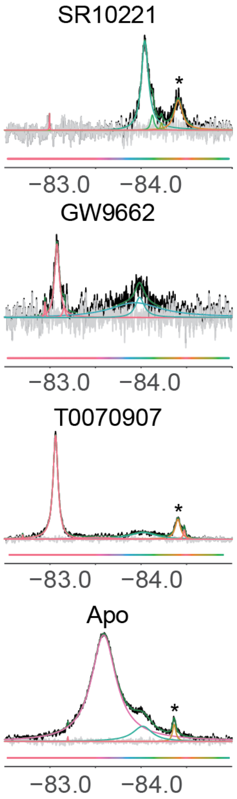


**Supplementary Figure 10. SMRT induces similar changes to helix 12 as NCoR.** A 2:1 molar ratio of SMRT peptide was added to ligand free PPARγ$^{K502C}$-BTFA (apo) or PPARγ$^{K502C}$-BTFA bound to the indicated ligands. Fluorine NMR was then performed.

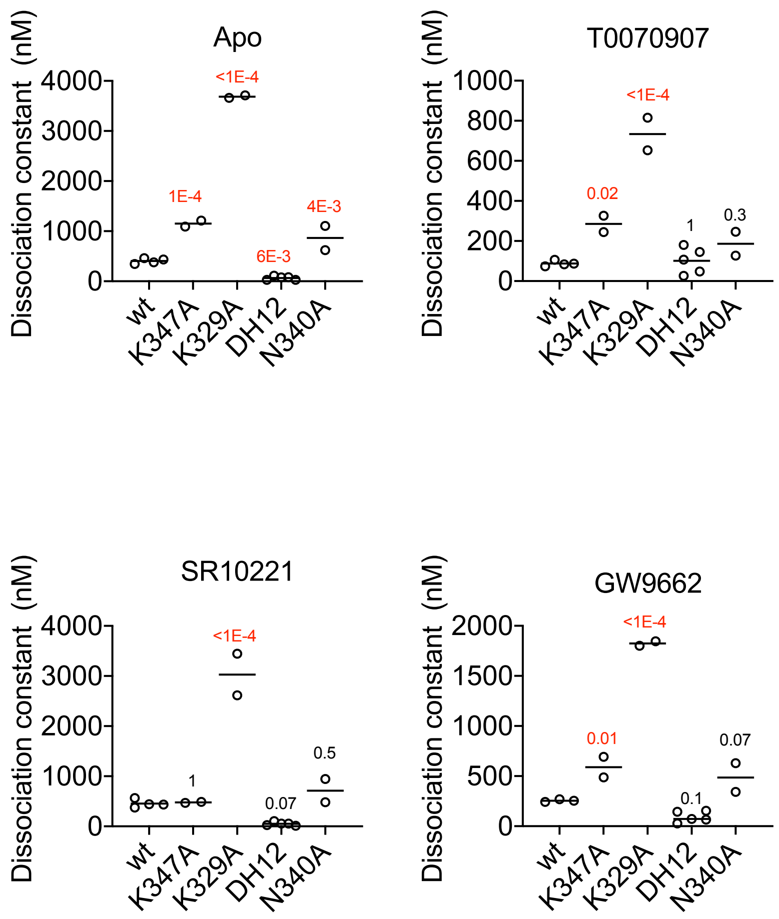


**Supplementary Figure 11. Affinity of various mutants vs wt PPARγ LBD for the corepressor NCoR either without ligand (apo) or bound to the indicated ligands.** P values, adjusted for multiple comparisons, are shown above the data points for comparison with wt. P values less than 0.05 are colored red. P values were determined by one-way ANOVA followed by Dunnett's multiple comparisons test between wt and the mutants. Fluorescein labeled coregulator peptide affinity was measured via fluorescence anisotropy. These data are also presented in Supplementary Table 3. All comparisons have 10 degrees of freedom except for GW9662 which has 9. Averages, mean difference ± SE of difference follow:

Apo averages: wt (405), K347A (1150), K329A (3687), DH12 (66), N340A (864); wt vs K347A difference=-745 ± 102; wt vs K329A difference=-3281±102; wt vs DH12 difference = 339 ± 79; wt vs N340A difference = -459 ± 102.

T0070907 averages: wt (88), K347A (286), K329A (734), DH12 (102), N340A (187); wt vs K347A difference=-198 ± 55; wt vs K329A difference=-646±55; wt vs DH12 difference = -14 ±43; wt vs N340A difference = -99 ± 55.

SR10221 averages: wt (454), K347A (478), K329A (3029), DH12 (50), N340A (713); wt vs K347A difference=-24 ± 189; wt vs K329A difference=-2574±189; wt vs DH12 difference = 403 ±146; wt vs N340A difference = -259 ± 189.

GW9662 averages: wt (255), K347A (590), K329A (1824), DH12 (93), N340A (485); wt vs K347A difference=-335 ± 83; wt vs K329A difference=-1569±83; wt vs DH12 difference = 161 ±67; wt vs N340A difference = -230 ± 83.

Source data are provided as a Source Data file (Source data_Heidari.xlsx).

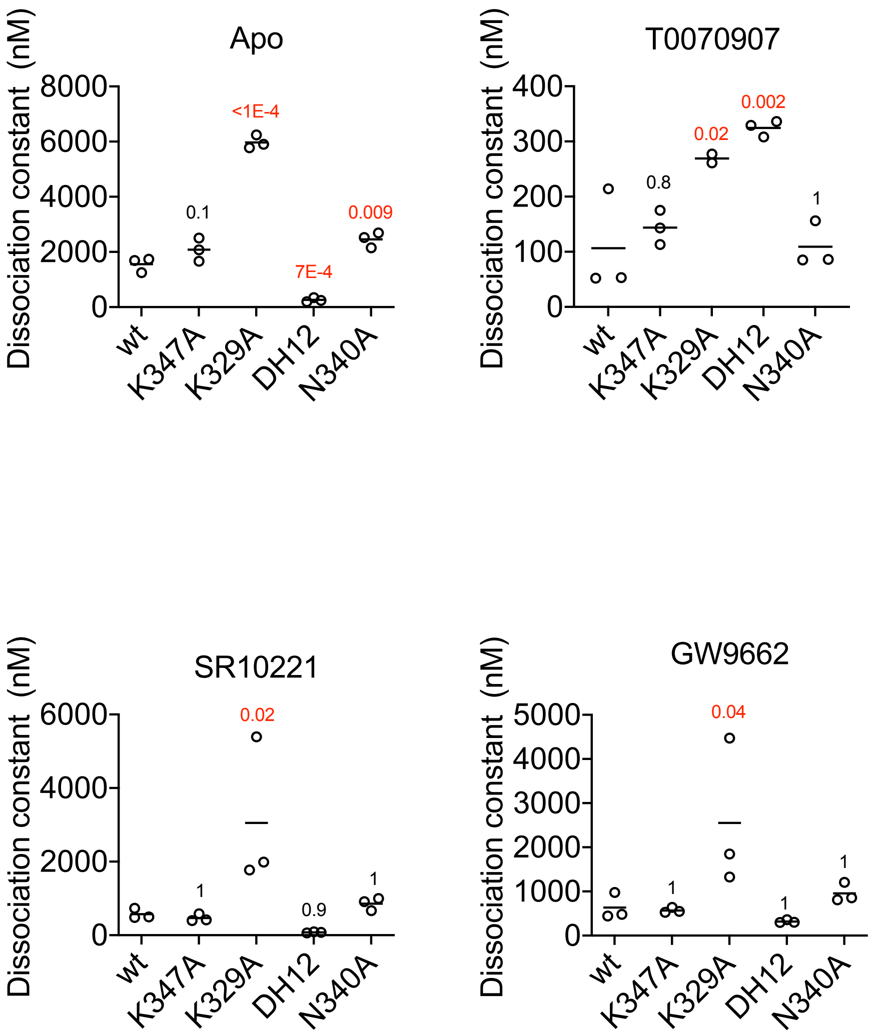


**Supplementary Figure 12. Affinity of various mutants vs wt PPARγ LBD for the corepressor SMRT either without ligand (apo) or bound to the indicated ligands.** P values, adjusted for multiple comparisons, are shown above the data points for comparison with wt. P values less than 0.05 are colored red. P values were determined by one-way ANOVA followed by Dunnett's multiple comparisons test between wt and the mutants. FITC labeled coregulator peptide affinity was measured via fluorescence anisotropy. These data are also presented in Supplementary Table 4. All comparisons have 10 degrees of freedom except for T0070907 which has 9. Averages, mean difference ± SE of difference follow:

Apo averages: wt (1557), K347A (2081), K329A (5973), DH12 (261), N340A (2454); wt vs K347A difference=-523 ± 228; wt vs K329A difference=-4416±228; wt vs DH12 difference = 1297 ± 228; wt vs N340A difference = -897 ± 228.

T0070907 averages: wt (106), K347A (144), K329A (269), DH12 (324), N340A (109); wt vs K347A difference=-37 ± 41; wt vs K329A difference=-163±46; wt vs DH12 difference = -218 ±41; wt vs N340A difference = -3 ± 41.

SR10221 averages: wt (570), K347A (465), K329A (3051), DH12 (77), N340A (858); wt vs K347A difference=105 ± 747; wt vs K329A difference=-2480±747; wt vs DH12 difference = 494 ±747; wt vs N340A difference = -287 ± 747.

GW9662 averages: wt (634), K347A (570), K329A (2550), DH12 (320), N340A (957); wt vs K347A difference=64 ± 631; wt vs K329A difference=-1916±631; wt vs DH12 difference = 314 ±631; wt vs N340A difference = -323 ±631.

Source data are provided as a Source Data file (Source data_Heidari.xlsx).

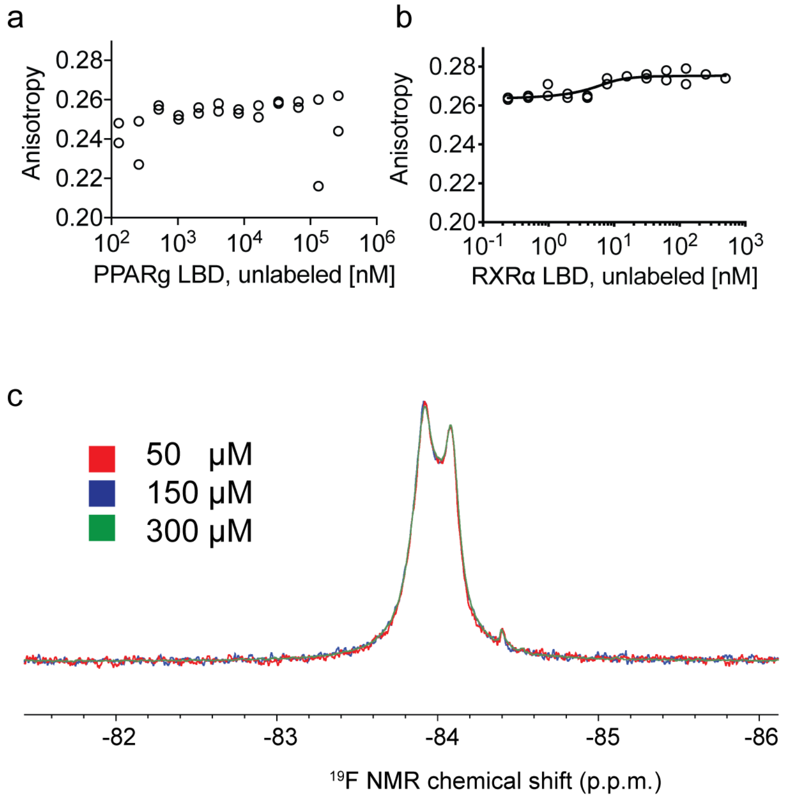


**Supplementary Figure 13. Apo PPARγ LBD is monomeric at the concentration used for NMR in this work (150 μM) and changing the concentration does not affect the spectrum.** a) The indicated concentrations of wt PPARγ LBD (129nM to 264μM) were added to 300nM PPARγ$^{Q373C,C313A}$-Hilyte488 and fluorescence anisotropy was measured as described in methods. Prism would not fit these data. b) The indicated concentrations of wt RXRα LBD (0.24nM to 500nM) were added to 8nM PPARγ$^{Q373C,C313A}$-Hilyte488 and fluorescence anisotropy was measured as described in methods. The data were fit as described in methods to yield a dissociation constant of 1nM (95% CI=0-6nM). The buffer used in these assays was 25mM MOPS, 25mM KCl, 1mM EDTA,0.01% Tween 20, 0.01% fatty acid free BTFA, pH 7.4. Individual technical replicates are shown for each point. c) Overlaid fluorine NMR spectra of 50, 150, and 300μM apo PPARγ$^{K502C}$-BTFA. The spectral intensities were adjusted for this overlay.

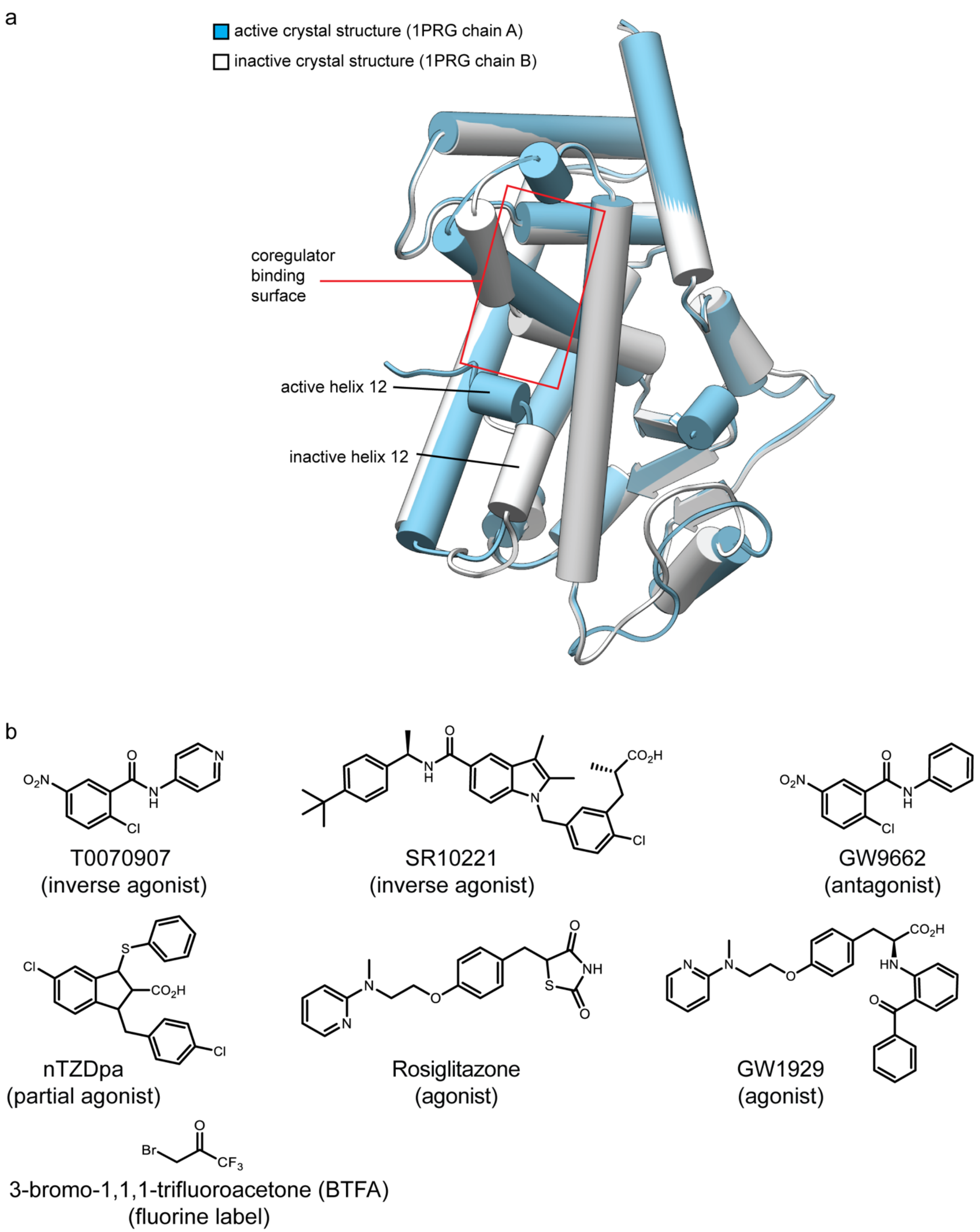


**Supplementary Figure 14. Active and inactive helix 12 conformations and illustration of the small molecules used in this study.** a) Overlay of the active (chain A) and inactive (chain B) chains of an apo crystal structure of PPARγ LBD (PDB code 1PRG) b) Small molecules used in this study.

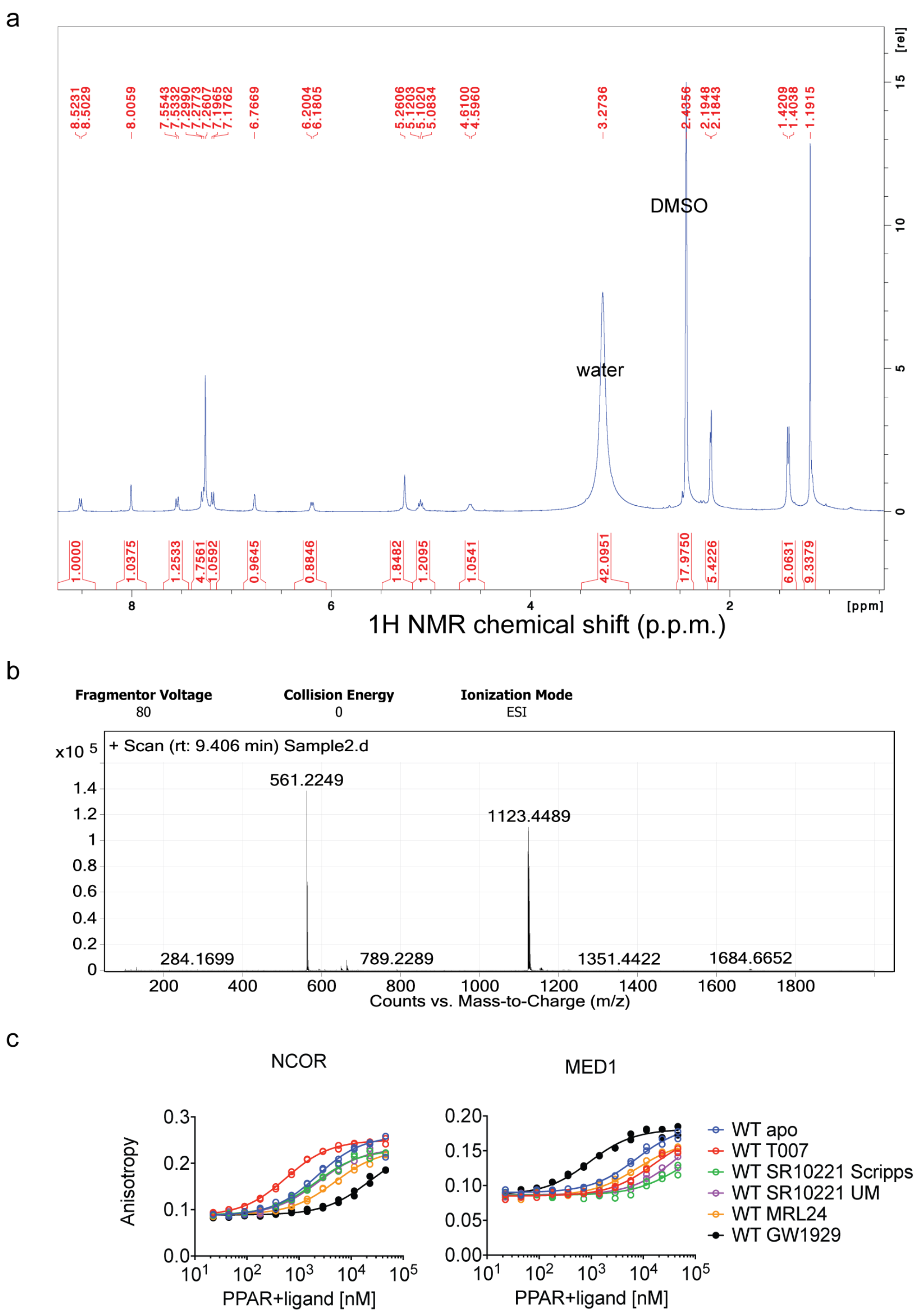


**Supplementary Figure 15. Comparison of SR10221 synthesized in different labs.** a) 1H proton NMR of SR10221 synthesized at the University of Montana and used in Figure 7d (see Supplementary Table 8 for comparison of spectrum with our previously reported spectrum for SR10221 produced at Scripps Florida[2]). The Scripps Florida produced SR10221 was used in all other figures. b) Electrospray ionization mass spectrometry of the SR10221 synthesized at the University of Montana. The mass of SR10221 is 562 daltons. A dimer species is also present, which is a known phenomenon for some compounds[3]. c) Comparison of the effect of SR10221 binding on the affinity of NCoR and MED1 peptides on PPARγ containing the LBD, hinge and DNA binding domain regions (amino acid residues 131-505). Data were fit as detailed in methods. 95% CI of dissociation constants for NCoR were apo (1.9-2.6μM), T0070907 (0.5-0.6μM), Scripps SR10221 (1.7-2.5μM), University of Montana SR10221 (2.0-2.8μM), MRL24 (4.2-5.7μM), GW1929 (15.6-32.4μM). 95% CI of dissociation constants for MED1 were apo (5.9-8.8μM), T0070907 (11.6-22.2μM), Scripps SR10221 (16.8-99.6μM), University of Montana SR10221 (23.4-49.2μM), MRL24 (5.8-10.4μM), GW1929 (0.7-1.2μM).

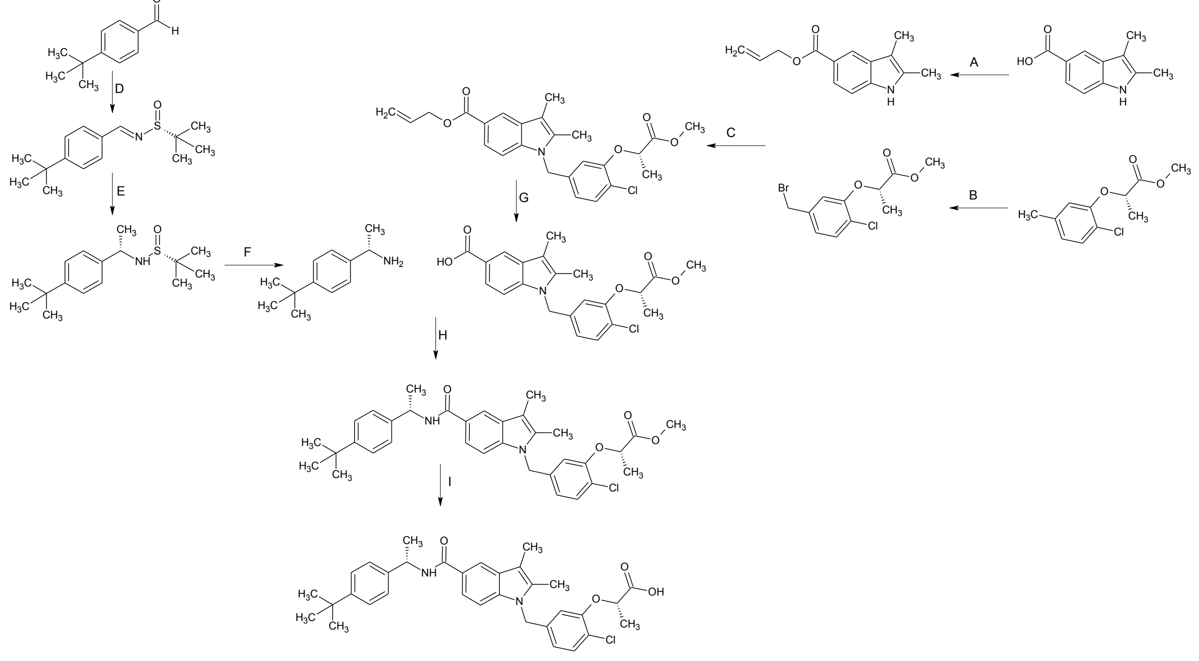


**Supplementary Figure 16. Overview of SR10221 synthesis at University of Montana.** (A) Allyl bromide, $K_2CO_3$, DMF, 3 hrs at rt. (B) NBS, AIBN, $CCl_4$, reflux overnight. (C) NaH, DMF, monitored by TLC until completion. (D) (R)-2-methylpropane-2-sulfinamide, PPTS, $MgSO_4$, DCM, reflux overnight. (E) MeMgBr, DCM, 6 hrs at -50°C, overnight at rt (F) HCl, MeOH, 30 min. at rt. (G) Morpholine, Pd($PPh_3$), THF, 1 hr at rt. (H) DIEA, HATU, DMF 15 hrs at rt. (I) NaOH, MeOH, THF, added DMSO until dissolved, 2 hrs at rt.

**Supplementary Table 1. Comparison of coregulator peptide affinity for the ligand binding domain of wt PPARγ LBD and PPARγ$^{Q299C}$-BTFA LBD of fluorescein labeled coregulator peptides measured via fluorescence anisotropy.**

| | WT $K_d$ (nM) | | PPARγ$^{Q299C}$-BTFA $K_d$ (nM) | | | |
|---|---|---|---|---|---|---|
| **DRUG** | NCoR | MED1 | NCoR | Fold Change (Q299C/WT) | MED1 | Fold Change (Q299C/WT) |
| NO LIGAND (APO) | 423 | 4988 | 490 | 1.2 | 2818 | 0.6 |
| T0070907 (INV. AGONIST) | 92 | 17286 | 80 | 0.9 | 18700 | 1.1 |
| GW9662 (ANTAGONIST) | 420 | 4902 | 318 | 0.8 | 3809 | 0.8 |
| SR1664 (ANTAGONIST) | 342 | 2965 | 316 | 0.9 | 3744 | 1.3 |
| MRL24 (PARTIAL AGONIST) | 827 | 2089 | 791 | 1.0 | 2489 | 1.2 |
| INT131 (PARTIAL AGONIST) | 1130 | 2602 | 1236 | 1.1 | 2503 | 1.0 |
| ROSIGLITAZONE (AGONIST) | 3636 | 512 | 2679 | 0.7 | 398 | 0.8 |
| GW1929 (AGONIST) | 3471 | 321 | 6127 | 1.8 | 339 | 1.1 |
| | | | median | 0.9 | median | 1.0 |
| | | | average | 1.0 | average | 1.0 |

**Supplementary Table 2. Comparison of coregulator peptide dissociation constants for the ligand binding domain of wt PPARγ, PPARγ$^{Q322C}$-BTFA, and PPARγ$^{C313A,Q322C}$-BTFA measured via fluorescence anisotropy.^**

| Ligand name (ligand type) | MED1 $K_d$ (nM) | | | | | | NCoR $K_d$ (nM) | | | | | |
|---|---|---|---|---|---|---|---|---|---|---|---|---|
| | wt | PPARγ C313A Q322C BTFA | FC* | wt | PPARγ Q322C BTFA | FC* | wt | PPARγ C313A Q322C BTFA | FC* | wt | PPARγ Q322C BTFA | FC* |
| Apo | 6340 | 10519 | 1.7 | 5799 | 3595 | 0.6 | 437 | 314 | 0.7 | 416 | 399 | 1.0 |
| T0070907# (inv. agonist) | 26646 | 7113 | 0.3 | 21558 | 9298 | 0.4 | 84 | 409 | 4.9 | 74 | 57 | 0.8 |
| GW9662# (antagonist) | 4218 | 9751 | 2.3 | | | | 273 | 348 | 1.3 | | | |
| SR1664 (antagonist) | 3916 | 6486 | 1.7 | | | | 281 | 404 | 1.4 | | | |
| MRL24 (partial agonist) | 2195 | 9145 | 4.2 | 3085 | 1760 | 0.6 | 884 | 1444 | 1.6 | 730 | 406 | 0.6 |
| SR10221 (inv. agonist) | | 14925 | | 19159 | 12172 | 0.6 | | | | | | |
| Int131 (partial agonist) | 1947 | 3964 | 2.0 | | | | 1218 | 1448 | 1.2 | | | |
| Troglitazone (agonist) | 1419 | 2444 | 1.7 | | | | 590 | 435 | 0.7 | | | |
| Pioglitazone (agonist) | 903 | 2468 | 2.7 | | | | 726 | 411 | 0.6 | | | |
| Rosiglitazone (agonist) | 305 | 1096 | 3.6 | 339 | 225 | 0.7 | 4395 | 1494 | 0.3 | 3239 | 4540 | 1.4 |
| GW1929 (agonist) | 224 | 1217 | 5.4 | 279 | 199 | 0.7 | 5082 | 57226 | 11.3 | 7708 | 6771 | 0.9 |
| Median | | | 2.2 | | | 0.6 | | | 1.1 | | | 0.8 |
| Mean | | | 2.6 | | | 0.6 | | | 1.9 | | | 0.9 |

*Fold change (FC) means labeled/wt dissociation constants.

#These ligands covalently bind to C313 and so are expected to be apo-like in PPARγ$^{C313A,Q322C}$-BTFA.

^The wt values were obtained in the same experiment for PPARγ$^{Q322C}$-BTFA but not for PPARγ$^{C313A,Q322C}$-BTFA.

**Supplementary Table 3. Dissociation constants between NCoR and PPARγ LBD alone or bound to the indicated ligands measured via fluorescence anisotropy. Number of separate experiments indicated by n.[#]**

| Construct | PPARγ (apo) nM±SD | PPARγ-T0070907 nM±SD (inverse agonist) | PPARγ-SR10221 nM±SD (inverse agonist) | PPARγ-GW9662 nM±SD (antagonist) |
|---|---|---|---|---|
| WT PPARγ | 405±54 n=4 | 88±14 n=4<br>*p=1E-5 | 454±80 n=4<br>*p=0.6<br>#p=3E-6 | 255±14 n=3<br>*p=0.01<br>#p=0.006<br>^p=0.002 |
| K329A (n=2) | 3687±35 | 734±115 | 3029±587 | 1824±32 |
| N340A (n=2) | 864±342 | 187±84 | 713±328 | 485±202 |
| K347A (n=2) | 1151±86 | 286±58 | 478±16 | 590±144 |
| Deletion of Helix 12 (n=5) | 66±37 | 102±65 | 50±37 | 93±55 |

[#]P value compared to *apo, #T0070907 and ^SR10221 was determined by one-way ANOVA followed by post-hoc analysis comparing all wt dissociation constants to each other using Dunnett's multiple comparisons test. All comparisons shown have 11 degrees of freedom. These data are also in Supplementary figure 11. For comparisons shown the mean difference ±SE follows: Apo vs T0070907 (317 ±36), Apo vs SR10221 (-49 ±36), Apo vs GW9662 (150 ±39), T0070907 vs SR10221 (-366 ±36), T0070907 vs GW9662 (-167 ±39), SR10221 vs GW9662 (199 ±39). Source data are provided as a Source Data file (Source data_Heidari.xlsx).

**Supplementary Table 4. Dissociation constants between SMRT peptide and wt PPARγ LBD or the indicated PPARγ LBD mutants measured via fluorescence anisotropy.**#**

| Construct | PPARγ (apo) nM±SD | PPARγ-T0070907 nM±SD (inverse agonist) | PPARγ-SR10221 nM±SD (inverse agonist) | PPARγ-GW9662 nM±SD (antagonist) |
|---|---|---|---|---|
| WT PPARγ (n=3) | 1557±270 | 106±93<br>*p=0.0002 | 570±142<br>*p=0.002<br>#p=0.1 | 634±297<br>*p=0.004<br>#p=0.07<br>^p=1 |
| K329A (n=3) | 5937±242 | 269±11@ | 3051±2031 | 2550±1687 |
| N340A (n=3) | 2454±278 | 109±41 | 858±171 | 957±216 |
| K347A (n=3) | 2081±418 | 144±31 | 465±103 | 570±59 |
| Deletion of Helix 12 (n=3) | 261±75 | 324±15 | 77±16 | 320±36 |

@ For PPARγ-T0070907 K329A one value was thrown out (5903 nM) both here and in the analysis in Supplementary Figure 12, therefore n=2 for this one mutant/ligand combination. This value was thrown out because it is highly unlikely to have come from T0070907 bound PPARγ and the other two values were consistent.

* P value compared to *apo, #T0070907 and ^SR10221 was determined by one-way ANOVA followed by post-hoc analysis comparing all wt dissociation constants to each other using Dunnett's multiple comparisons test.

**These data are also in Supplementary Figure 12. Source data are provided as a Source Data file (Source data_Heidari.xlsx).

#All comparisons shown have 9 degrees of freedom and the mean difference ±SE follows: Apo vs T0070907 (1451 ±178), Apo vs SR10221 (987 ±178), Apo vs GW9662 (923 ±178), T0070907 vs SR10221 (-464 ±178), T0070907 vs GW9662 (-528 ±178), SR10221 vs GW9662 (-64 ±178).

**Supplementary Table 5. Duration of aMD simulations^**

| *PPARγ ligand binding domain complex* | Total length of simulations (μs) | number of independent runs | Starting structure (original equilibrated structure / from long cMD simulation)# |
|---|---|---|---|
| *apo-NCoR* | 11.9 | 5 | 2 / 3 |
| *apo* | 56.25 | 7 | 2 / 5 |
| **active apo* | 12.75 | 6 | 4 / 2 |
| *T0070907-NCoR* | 13.5 | 6 | 4 / 2 |
| *T0070907* | 15.75 | 7 | 4 / 3 |
| *GW9662-NCoR* | 10.8 | 5 | 2 / 3 |
| *GW9662* | 8.1 | 6 | 2 / 4 |
| *SR10221-NCoR* | 11.25 | 5 | 2 / 3 |
| *SR10221* | 9 | 4 | 2 / 2 |
| **rosiglitazone* | 9 | 4 | 2 / 2 |

**started with helix 12 in an active conformation*
*# These numbers indicate the source of the starting structure for the independent aMD simulations. Some were started from the minimized and equilibrated structure with distinct atomic velocities (first number), while others were started from distinct structures produced through conventional MD runs (second number).*
*^ Source data are provided as a Source Data file (Source data_Heidari.xlsx).*

**Supplementary Table 6. Solvation of NCoR residues in wt and mutant PPARγ-NCoR complexes bound to the indicated ligands.**

| | carbonyl O of NCoR residue A2270 | | | Arginine side chain of NCoR residue R2268 | | |
|---|---|---|---|---|---|---|
| | wt | N340A | N340A-wt | wt | K329A | K329A-wt |
| **T0070907** | 1.2422 | 2.1903 | 0.95 | 7.2963 | 7.7423 | 0.45 |
| **GW9662** | 1.1333 | 2.1412 | 1.00 | 7.3156 | 7.4256 | 0.11 |
| **SR10221** | 1.1846 | 1.8381 | 0.65 | 7.1038 | 7.6183 | 0.51 |
| **apo** | 1.44918 | 2.2088 | 0.76 | 7.3769 | 7.3099 | -0.07 |

**Supplementary Table 7. Energy Values of minimums in the potential energy landscape.**

| *energy level* | 0 | 1 | 2 | 3 | 4 | 5 |
|---|---|---|---|---|---|---|
| | (kcal $mol^{-1}$ relative to well with lowest energy) | | | | | |
| *apo PPARγ-NCoR* | 0 | 0.25 | 0.40 | 0.42 | 0.47 | 0.56 |
| *PPARγ-SR10221-NCoR* | 0 | 0.20 | 0.80 | | | |
| *PPARγ-GW9662-NCoR* | 0 | 0.20 | 1.61 | 1.61 | | |
| *PPARγ-T0070907-NCoR* | 0 | 0.35 | 0.46 | 0.47 | 0.55 | |
| *apo PPARγ (active)* | 0 | 0.09 | 1.45 | 1.58 | 1.76 | |
| *apo PPARγ (inactive)* | 0 | 0.75 | 1.06 | 1.12 | 1.23 | |
| *PPARγ-GW9662* | 0 | 0.09 | | | | |
| *PPARγ-T0070907* | 0 | 0.10 | 0.69 | | | |
| *PPARγ-rosiglitazone* | 0 | | | | | |
| *PPARγ-SR10221* | 0 | 0.34 | 1.04 | | | |

## Supplementary Table 8. Comparison of UM and Scripps synthesized SR10221 using 1H 400 Mhz NMR.

| | University of Montana synthesized SR10221 | | | | | | | Scripps SR10221 (from Marciano et. al. http://www.ncbi.nlm.nih.gov/pubmed/26068133) | | | | |
|---|---|---|---|---|---|---|---|---|---|---|---|---|
| | observed peaks (p.p.m.) | | | | | J coupling (Hz) | int. | type | reported peaks (p.p.m.) | reported J coupling (Hz) | reported integration | Difference between observed and reported chemical shifts |
| | 8.523 | 8.503 | | | | 8.10 | 1.0 | d | 8.57 | 8.1 | 1 | -0.0469 |
| | 8.006 | | | | | unresolved | 1.0 | d | 8.08 | 1.5 | 1 | -0.0741 |
| | 7.554 | 7.533 | | | | unresolved, 8.4 | 1.3 | dd | 7.6 | 1.8,8.6 | 1 | -0.0457 |
| | 7.299 | 7.277 | | | | 8.68 | 4.8 | d | 7.36 | 8.6 | 1 | -0.061 |
| | 7.261 | | | | | unresolved | | multiplet | 7.33 | NA | 3 | -0.0693 |
| | 7.197 | 7.176 | | | | 8.12 | 1.1 | d | 7.29 | 8.1 | 1 | -0.0935 |
| | 6.767 | | | | | unresolved | 1.0 | d | 6.79 | 1.8 | 1 | -0.0231 |
| | 6.200 | 6.181 | | | | unresolved, 7.96 | 0.9 | dd | 6.34 | 1.8,8.1 | 1 | -0.1396 |
| | 5.261 | | | | | NA | 1.8 | s | 5.47 | | 2 | -0.2094 |
| | 5.138 | 5.120 | 5.102 | 5.083 | 5.068 | not calculated | 1.2 | quintuplet | 5.17 | 7.1 | 1 | -0.0316 |
| | 4.610 | 4.596 | | | | 5.60 | 1.1 | quintuplet | 4.78 | 6.8 | 1 | -0.17 |
| | 2.195 | | | | | NA | 5.4 | s | 2.27 | | 3 | -0.0752 |
| | 2.184 | | | | | NA | | s | 2.26 | | 3 | -0.0757 |
| | 1.421 | | | | | unresolved | 6.1 | d | 1.5 | 6.8 | 3 | -0.0791 |
| | 1.404 | | | | | unresolved | | d | 1.47 | 7.1 | 3 | -0.0662 |
| | 1.192 | | | | | NA | 9.3 | s | 1.26 | | 9 | -0.0685 |
| | | | | | | | | | Commonly reported value in D6-DMSO | | | |
| **water** | 3.274 | | | | | | 42.1 | | 3.3 | | | -0.026 |
| **DMSO** | 2.436 | | | | | | 18.0 | | 2.5 | | | -0.064 |

**Supplementary Table 9. Summary of structural and functional evidence.***

| EFFECT OF LIGAND BINDING ON STRUCTURE OF: | T0070907 (inverse agonist) | SR10221 (biased inverse agonist) | GW9662 (antagonist) | Apo/vehicle | Rosiglitazone (agonist) |
|---|---|---|---|---|---|
| HELIX 12 | two states in slow exchange 70% state 1 (highly repressive; low local disorder) 30% state 2 (similar to apo; high local disorder) | multiple displaced and/or disordered states; high local disorder | two states in slow exchange 10% state 1 (highly repressive) 90% state 2 (similar to apo) | A complex mixture of states, including autorepressed, displaced and active states; most states have high local disorder | The canonical active state; low local disorder |
| HELIX 3 CHARGE CLAMP | one primary state with low local disorder | medium local disorder | medium local disorder | A complex mixture of states including large movements and disrupted helicity resulting in large movements of the n-terminal half of helix 3 | one primary state with low local disorder |
| OMEGA LOOP | two states in slow exchange 70% state 1 (highly repressive; low local disorder) 30% state 2 (multiple conformations) | multiple conformations | two states in slow exchange 10% state 1 (highly repressive) 90% state 2 (multiple conformations) | two states in slow exchange 10% state 1 (highly repressive) 90% state 2 (multiple conformations) | multiple conformations |
| **EFFECT OF LIGAND BINDING ON:** | | | | | |
| COREPRESSOR CORNR BOX PEPTIDE AFFINITY | ↑↑ | ↑ SMRT no effect on NCoR | ↑ | Comparator | ↓↓ |
| COACTIVATOR LXXLL BOX PEPTIDE AFFINITY | ↓↓ | ↓↓ | no effect | Comparator | ↑↑ |
| REPORTER EXPRESSION IN CELLS | ↓↓ | ↓↓ | no effect | Comparator (likely is bound with fatty acid partial agonists) | ↑↑ |

*Structural and functional effects presented here are based on data reported here and previously[1,4]

Supplementary Table 10. Primers used for mutations.

| *Mutation* | Primer sequence |
|---|---|
| *C313A* | 5'-ccacggagcgaaactgagcgccctgaaagatgcgg-3' |
| *Q322C* | 5'-gcatactctgtgatctcgcacacagcctccacggagc-3' |
| *K329A* | 5'-tttacaaaaccaggaatgcttgcggcatactctgtgatctcctg-3' |
| *N340A* | 5'-atttgaggagagttacttggtcggccaagtcaagatttacaaaaccag-3' |
| *K347A* | 5'-gatctcgtggactccatatgcgaggagagttacttggtcg-3', |
| *ΔH12* | 5'-tccttgtagatctcctgcagttacgggtgaagactcatgtctg-3' |

Supplementary References

1. Chrisman, I. M., Nemetchek, M. D., de Vera, I. M. S., Shang, J., Heidari, Z., Long, Y., Reyes-Caballero, H., Galindo-Murillo, R., Cheatham, T. E., Blayo, A.-L., Shin, Y., Fuhrmann, J., Griffin, P. R., Kamenecka, T. M., Kojetin, D. J. & Hughes, T. S. Defining a conformational ensemble that directs activation of PPARγ. *Nat. Commun.* **9,** 1794 (2018).
2. Marciano, D. P., Kuruvilla, D. S., Boregowda, S. V., Asteian, A., Hughes, T. S., Garcia-Ordonez, R., Corzo, C. A., Khan, T. M., Novick, S. J., Park, H., Kojetin, D. J., Phinney, D. G., Bruning, J. B., Kamenecka, T. M. & Griffin, P. R. Pharmacological repression of PPARγ promotes osteogenesis. *Nat. Commun.* **6,** 7443 (2015).
3. De Vijlder, T., Valkenborg, D., Lemière, F., Romijn, E. P., Laukens, K. & Cuyckens, F. A tutorial in small molecule identification via electrospray ionization-mass spectrometry: The practical art of structural elucidation. *Mass Spectrom. Rev.* **37,** 607–629 (2018).
4. Brust, R., Shang, J., Fuhrmann, J., Mosure, S. A., Bass, J., Cano, A., Heidari, Z., Chrisman, I. M., Nemetchek, M. D., Blayo, A.-L., Griffin, P. R., Kamenecka, T. M., Hughes, T. S. & Kojetin, D. J. A structural mechanism for directing corepressor-selective inverse agonism of PPARγ. *Nat. Commun.* **9,** 4687 (2018).
