# Supplementary material for: Definition of functionally and structurally distinct repressive states in the nuclear receptor PPARγ
Source: Nat Commun. 2019 Dec 20;10:5825. doi: 10.1038/s41467-019-13768-0 (PMC6925260; doi:10.1038/s41467-019-13768-0)
Supplement: Supplementary file 2 — Reporting Summary [file 41467_2019_13768_MOESM2_ESM.pdf]

nature research

Corresponding author(s): Travis Hughes
Last updated by author(s): 11/15/2019

Reporting Summary

Nature Research wishes to improve the reproducibility of the work that we publish. This form provides structure for consistency and transparency in reporting. For further information on Nature Research policies, see Authors & Referees and the Editorial Policy Checklist.

Statistics

For all statistical analyses, confirm that the following items are present in the figure legend, table legend, main text, or Methods section.

n/a	Confirmed	
☐	☒	The exact sample size (n) for each experimental group/condition, given as a discrete number and unit of measurement
☐	☒	A statement on whether measurements were taken from distinct samples or whether the same sample was measured repeatedly
☐	☒	The statistical test(s) used AND whether they are one- or two-sided<br>*Only common tests should be described solely by name; describe more complex techniques in the Methods section.*
☒	☐	A description of all covariates tested
☐	☒	A description of any assumptions or corrections, such as tests of normality and adjustment for multiple comparisons
☐	☒	A full description of the statistical parameters including central tendency (e.g. means) or other basic estimates (e.g. regression coefficient) AND variation (e.g. standard deviation) or associated estimates of uncertainty (e.g. confidence intervals)
☐	☒	For null hypothesis testing, the test statistic (e.g. F, t, r) with confidence intervals, effect sizes, degrees of freedom and P value noted<br>*Give P values as exact values whenever suitable.*
☒	☐	For Bayesian analysis, information on the choice of priors and Markov chain Monte Carlo settings
☒	☐	For hierarchical and complex designs, identification of the appropriate level for tests and full reporting of outcomes
☒	☐	Estimates of effect sizes (e.g. Cohen's d, Pearson's r), indicating how they were calculated

Our web collection on statistics for biologists contains articles on many of the points above.

Software and code

Policy information about availability of computer code

Data collection	NMR: Bruker Topspin 3.5 was used to acquire data and version 4.0 was used for processing and analysis.<br>BioTek Gen5 version 3.00.19 was used to acquire anisotropy data.<br>HDX-MS HDXWorkbench software was used.<br>AmberTools 14 was used to carry out simulations.<br>Chimera version 1.13.1 build 41965 along with earlier versions (unknown exactly which versions) were used to build simulation complexes.<br>As described in methods the H++ server (http://biophysics.cs.vt.edu/H++) and RED server (http://upjv.q4md-forcefieldtools.org/REDServer-Development/) were used to make simulation builds.
Data analysis	NMR Spectra were deconvoluted objectively with models chosen statistically by an open source fitting program described in our publication "Deconvolution of Complex 1D NMR Spectra Using Objective Model Selection" PLoS one (DOI 10.1371/journal.pone.0134474)<br>Chimera version 1.13.1 build 41965 along with earlier versions (unknown exactly which versions) were used to analyze simulations and crystal structures.<br>We used Graphpad Prism version 8.1.2 to do all statistical analysis and fitting of dissociation constants. For fitting of dissociation constants we used a combination of equations from Biochemistry 43, 16056–16066 (2004) as described in detail in methods.<br>AmberTools 14 was used to analyze simulations. PyReweighting was used to generate native energy landscapes.<br>HDX-MS HDXWorkbench software was used.

For manuscripts utilizing custom algorithms or software that are central to the research but not yet described in published literature, software must be made available to editors/reviewers. We strongly encourage code deposition in a community repository (e.g. GitHub). See the Nature Research guidelines for submitting code & software for further information.

Data

Policy information about availability of data

All manuscripts must include a data availability statement. This statement should provide the following information, where applicable:

- Accession codes, unique identifiers, or web links for publicly available datasets
- A list of figures that have associated raw data
- A description of any restrictions on data availability

Raw data for the anisotropy experiments is publicly available at https://osf.io/kjbam/. Any other datasets generated during and/or analyzed during the current study are available from the corresponding author on reasonable request.

Field-specific reporting

Please select the one below that is the best fit for your research. If you are not sure, read the appropriate sections before making your selection.

☒ Life sciences ☐ Behavioural & social sciences ☐ Ecological, evolutionary & environmental sciences

For a reference copy of the document with all sections, see nature.com/documents/nr-reporting-summary-flat.pdf

Life sciences study design

All studies must disclose on these points even when the disclosure is negative.

Sample size	No sample size calculation was performed. We did two technical replicates and two or more experimental replicates for anisotropy experiments (see replication below). Many anisotropy experiments were done just twice to get a minimal idea of repeatability.
Data exclusions	All data exclusions are highlighted in the legend or were excluded objectively as stated in the methods. Objective exclusion was performed in Graphpad Prism for all of the dissociation constant fits performed as described in methods. The one case where we subjectively excluded a value was for one T0070907/K329A value in Supplementary Figure 12 and Supplementary Table 4 because it was very different from the other two values and not characteristic at all of T0070907 bound to PPARγ. This is stated in Supplementary Table 4 and the excluded value is also shown there.
Replication	We performed independent replicate experiments where we deemed it necessary. Much of the NMR was only run once, but some was run more than once and those replicates are presented. We have included all replications of data presented in the paper that we are not reporting in other papers. We have performed some similar coregulator peptide affinity measurement experiments and NMR that are not presented here because we are using them in an independent paper. These replicates show similar results to those presented here. For most experiments all samples were identical except for the variable under consideration (no covariants). There could be unknown covariates in the comparisons of affinity for coregulator peptides between wild type and mutant proteins using fluorescence anisotropy. This is because these experiments require using protein from independent purifications from bacteria. Because of this the mutants or wild-type proteins undoubtedly vary in the exact amount of co-purifying bacterial proteins, lipids etc. For comparison between wt and mutants, in order to determine if the mutation or any of these covariates were the cause of the changes in affinity observed we performed replicate experiments using protein from multiple independent protein purifications of wt and mutant (these were the replicates in the statistical comparisons).<br>We have performed a set of two fluorescence anisotropy experiments using E304L that we did not include in this paper. These data did not test SR10221, and because of this and the fact that we are planning on using these data in a future paper we did not include these data here. These data show again that the mutation increases affinity for NCoR across apo and several ligands (T0070907, partial agonists and full agonists) and decrease it for CBP. The relative change was nominally smallest for the T0070907 complex and apo for NCoR (as shown in the presented data). However all the complexes had relatively larger reductions (compared to the data presented here) in affinity for CBP, with partial and full agonists changing the least in CBP affinity.<br>For simulations, we did at least 4 independent replicate accelerated aMD simulations (see supplementary Table 5). We ran inactive apo simulations significantly longer than the others because we thought better convergence was reachable with this complex with a longer total simulation time based on the 19F NMR. As described in methods the aMDs were all combined to generate energy landscapes and then cMD simulations were started based on the aMD energy landscapes. The number of cMDs started depended on the number of low energy regions in the aMD generated energy landscape.
Randomization	We did not randomize any samples.
Blinding	Blinding was not performed because data collected was objective and not subjective.

Reporting for specific materials, systems and methods

We require information from authors about some types of materials, experimental systems and methods used in many studies. Here, indicate whether each material, system or method listed is relevant to your study. If you are not sure if a list item applies to your research, read the appropriate section before selecting a response.

Materials & experimental systems

n/a	Involved in the study	
☒	☐	Antibodies
☒	☐	Eukaryotic cell lines
☒	☐	Palaeontology
☒	☐	Animals and other organisms
☒	☐	Human research participants
☒	☐	Clinical data

Methods

n/a	Involved in the study	
☒	☐	ChIP-seq
☒	☐	Flow cytometry
☒	☐	MRI-based neuroimaging
